# A Dual-Mode Surface Display System for the Maturation and Production of Monoclonal Antibodies in Glyco-Engineered *Pichia pastoris*

**DOI:** 10.1371/journal.pone.0070190

**Published:** 2013-07-10

**Authors:** Hussam H. Shaheen, Bianka Prinz, Ming-Tang Chen, Tej Pavoor, Song Lin, Nga Rewa Houston-Cummings, Renee Moore, Terrance A. Stadheim, Dongxing Zha

**Affiliations:** GlycoFi, Biologics Discovery, Merck Research Laboratories, Merck & Co., Inc., Lebanon, New Hampshire, United States of America; National Cancer Institute, NIH, United States of America

## Abstract

State-of-the-art monoclonal antibody (mAb) discovery methods that utilize surface display techniques in prokaryotic and eukaryotic cells require multiple steps of reformatting and switching of hosts to transition from display to expression. This results in a separation between antibody affinity maturation and full-length mAb production platforms. Here, we report for the first time, a method in Glyco-engineered *Pichia* *pastoris* that enables simultaneous surface display and secretion of full-length mAb molecules with human-like N-glycans using the same yeast cell. This paradigm takes advantage of homo-dimerization of the Fc portion of an IgG molecule to a surface-anchored "bait" Fc, which results in targeting functional “half” IgGs to the cell wall of *Pichia* *pastoris* without interfering with the secretion of full length mAb. We show the utility of this method in isolating high affinity, well-expressed anti-PCSK9 leads from a designed library that was created by mating yeasts containing either light chain or heavy chain IgG libraries. Coupled with Glyco-engineered *Pichia* *pastoris* , this method provides a powerful tool for the discovery and production of therapeutic human mAbs in the same host thus improving drug developability and potentially shortening the discovery time cycle.

## Introduction

The use of surface display for discovery of novel monoclonal antibodies (mAbs) has evolved dramatically over the last decade. Multiple antibody display formats have been proposed and implemented in both prokaryotic and eukaryotic systems [1–5]. Despite these advances, the majority of these efforts have focused on the utilization of antibody fragments as surrogates for mAb lead identification and maturation rather than full-length immunoglobulin G proteins (IgGs). While the antigen binding domains of antibodies can be used to evolve binding affinity, they poorly predict the physicochemical characteristics and expressibility within the context of the full-length cognate IgGs. In addition, these approaches necessitate conversion and reformatting in order to express the identified leads in the preferred production hosts [6,7]. These additional steps account for delayed timelines and result in lead attrition due to the unpredictable differences between formats, and switching between display and expression hosts.

Recent reports have described novel platforms dedicated for the discovery of full-length IgGs in bacterial, mammalian, and yeast cell lines [1,8,9]. To date, even with extensive engineering, bacterial systems are unable to generate fully human glycosylated proteins, and thus require additional conversion steps for expression and selection of the discovered leads in mammalian cell lines. Furthermore, while bacterial display has been successful in isolating mAbs which could bind with nano-molar affinities to their desired target [10–12], the prokaryotic expression may not predict how proteins would behave once produced in the eukaryotic hosts that possess post-translational modifications such as glycosylation. In the case of mammalian display, direct anchoring of the IgG to the cell membrane was required [9]. This entailed the introduction of an additional ectopic sequence to the IgG heavy chain thus leading to additional conversion steps and further selection to enable soluble expression of full-length mAbs. Surface re-capture technologies for full-length mAbs in yeast were developed to enable display and secretion in the same cell. These methods utilized post-secretory binding of IgG molecules in the culture medium to an anchored moiety on the cell surface [13,14]. Binding was achieved directly through protein–protein or protein-ligand interactions. Although re-capture technologies couple secretion to display for full length IgGs, they suffer drawbacks of their own, including the need for modifying the protein sequence to allow surface binding in particular cases. Moreover, re-capture following secretion introduces the risk of "crosstalk" between clones that could lead to the loss of the required genotype-to-phenotype relationship that is critical for efficient lead-cell isolation. Without a strong genotype-to-phenotype relationship built into the display system the issue of "cross talk" among clones in the same culture must be addressed through tedious experimental modifications [15].

Until very recently, no methodology has been reported that utilizes cell surface display to facilitate discovery of full-length mAbs that unify discovery and production hosts while preserving the molecular integrity of the IgG [16]. Glyco-engineered *Pichia* *pastoris* has been successfully used for production of monoclonal antibodies and therapeutic development [17–20]. Here, we describe a dual-mode technique for engineering and production of full-length mAbs in Glyco-engineered *Pichia* *pastoris* . This approach preserves co-secretion of full-length unmodified bivalent mAbs into culture medium while utilizing the natural antibody biogenesis pathway to covalently display monovalent functional half IgG molecules on the cell wall. This facilitates the simultaneous selection of an antibody sequence with desired binding properties and its subsequent production in the same host. In addition, we also describe the first attempt to utilize this approach alongside haploid yeast mating by combining full-length heavy chain and full-length light chain libraries and the application of this display system to mature the expressibility of a monoclonal antibody lead while maintaining its affinity.

## Materials and Methods

### Strains, reagents, plasmids and *Pichia* transformation

DNA recombination and cloning were performed with restriction endonucleases and DNA modification enzymes from New England BioLabs (Beverly, MA) and *E. coli* strain TOP10 from Invitrogen (Carlsbad, CA). Oligonucleotides were synthesized by Integrated DNA Technologies (Coralville, IA) and PCR reactions were performed with *pfu*Turbo from Stratagene (La Jolla, CA). GPI protein genes were codon optimized for *P* *. pastoris* and synthesized by GeneArt AG (Regensburg, Germany). *Pichia* transformations were conducted using electroporation. Table 1 lists the yeast strains used in this study. All Glyco-engineered *Pichia* *pastoris* expression strains used were constructed from wild-type *Pichia* *pastoris* strain NRRL-Y11430 (Northern Regional Research Laboratories, Peoria, IL) using methods described in [21–24]. Anti-PCSK9 yeast display mating library construction was described in Chen *et al.* [25].

**Table 1:** Strains used in this study.

**Strain**	**Genotype**	**Production Protein**
YGLY8316	*och1Δ, bmt1Δ, bmt2Δ, bmt3Δ, bmt4Δ, mnn4L1Δ, pno1Δ, mnn4Δ,* *Kluyveromyces* *lactis* *& Mus musculus UDP-GlcNAc transporters,* *Trichoderma* *reesei* *α-1,2-MnsI, M. musculus α-1,2-MnsI,Homo sapiens β-1,2-GlcNAc transferase I, Rattus norvegicus β-1,2-GlcNAc transferase II, Drosophila melanogaster MnsII, Saccharomyces cerevisiae* Gal* epimerase, D. melanogaster UDP-Gal transporter, H. sapiens β-1,4-galactosyl transferase*	Empty parent and control
YGLY13979	YGLY8316 TRP2::AOX1p-anti-Her2-LcAOX1p- anti-Her2-Hc ZeoR	Anti-Her2
YGLY18483	YGLY8316 TRP2::AOX1p-anti-PCSK92-LcAOX1p-anti-PCSK9-Hc ZeoR	Anti-PCSK9

### Construction and co-expression of Fc-Sed1p expression cassette

To create the plasmid containing the Fc bait cassette, a codon optimized sequence of human IgG1 Fc fragment was synthesized using an EcoRI forward PCR primer containing the nucleic acid sequence of *S. cerevisiae* α-mating factor signal sequence fused upstream of the sequence encoding the IgG1 Fc N-terminus (DKTHTCPPC.), and a SalI reverse primer encoding the C-terminus of IgG1 Fc that terminates in a sequence encoding a GGGG linker. A plasmid containing the human IgG1 heavy-chain gene sequence was used as a PCR template for amplification of an EcoRI-α-mating factor signal sequence-Fc-GGGG-SalI fragment. Both PCR product and pGLY3033 [3] were digested using EcoRI and SalI endonucleases. The EcoRI-SalI fragment encoding the Fc was ligated in frame to EcoRI-SalI pGLY3033 backbone to generate plasmid pGLY9008. This plasmid enables delivery of the *FC-ScSED1* cassette under the control of the *Pichia* *pastoris* *AOX1* promoter sequence. Like the parent plasmid, it contains the *Pichia* *pastoris* *URA6* gene sequence, which serves as an integration locus in the genome, and the arsenite resistance gene, to allow selection on media containing sodium arsenite.

### Bioreactor Cultivations -1 Liter and Micro24 (4 mL) Cultivations

One Liter Bioreactor and Micro24 cultivations were performed as described previously [25].

### Antigen binding of anti-PCSK9 antibodies

The binding affinity of the anti-PCSK9 antibodies was measured on a Biacore T100 instrument with a carboxymethylated dextran (CM5, cat# BR-1006-68) chip and 1× HBS-EP+ (10 mM HEPES, 150 mM NaCl, 3 mM EDTA, and 0.05% Surfactant P20) as the running buffer. The CM5 chip was immobilized on all flow cells with mouse anti-human IgG (Fc specific) according to the Biacore Human Antibody Capture Kit (Cat# BR-1008-39) to ~ 7000 RU. Anti-PCSK9 antibodies were captured on the chip to ~ 500 RU followed by analyte injections of wild-type human PCSK9 from 0.156 nM to 2.5 nM, except for the 96-well affinity measurements were crude supernatants were captured followed by a single injection of 2.5 nM of rhPCSK9. Each flowcell was regenerated between each analyte injection with 3 M MgCl for 40 s at 10 μl/min. Data was analyzed with Biacore T100 Evaluation Software using the 1/1 binding model.

### Cell labeling

After induction on methanol, 2 OD_600_ of cells(~ 10^7^ cells) were collected into a 1.5-ml microfuge tube and washed twice with phosphate-buffered saline (PBS, Sigma, St. Louis, MO) and then suspended in 100 μl of PBS containing 1 μl (2 μg) of goat anti-human Fc DyeLight 488, or anti-human Kappa light chain APC 635 (Invitrogen, Carlsbad, CA) at room temperature for 30 min. When labeling for both expression and affinity, PCSK9 conjugated with Biotin (Merck, Whitehouse Station, NJ) was also added at a final concentration of 20 nm and detected with Streptavidin conjugated with DyeLight 488 or APC 635. After incubation with detection antibodies/reagents the cells were washed twice with PBS and suspended in 100 μl of PBS for flow cytometric analysis and sorting (when required). The labeled cells were kept on ice and protected from light throughout the experiment.

### Flow cytometric analysis and cell sorting

Flow cytometric analysis and cell sorting were performed on a FACSAria cell sorter with blue and red lasers (BD Biosciences, San Jose, CA) equipped with FACSDiva software. The procedure was performed according to Lin et al. [3]. Gating in a dot plot of FSC vs. SSC was routinely applied to exclude cell debris and to include a population of single cells with similar size for analysis and sorting. For each sort, the 1% of cells with the brightest signal were gated and 5000–10,000 total cells were collected. Flow cytometry data were analyzed with software FlowJo v 7.1.2 (Tree Star, Inc., Ashland, OR).

### Purification of mAbs

mAbs were purified using a one-step, 96-well format STREAMLINE rProtein A methods described in [26].

### Protein electrophoresis

Proteins were resolved using LabChip® GX II (PerkinElmer, Waltham, MA).

### Glycan characterization

PNGase F, 2-aminobenzamide (2-AB) labeling and high performance liquid chromatography (HPLC) analysis of glycans has been described previously [22].

## Results

**Figure 1:**
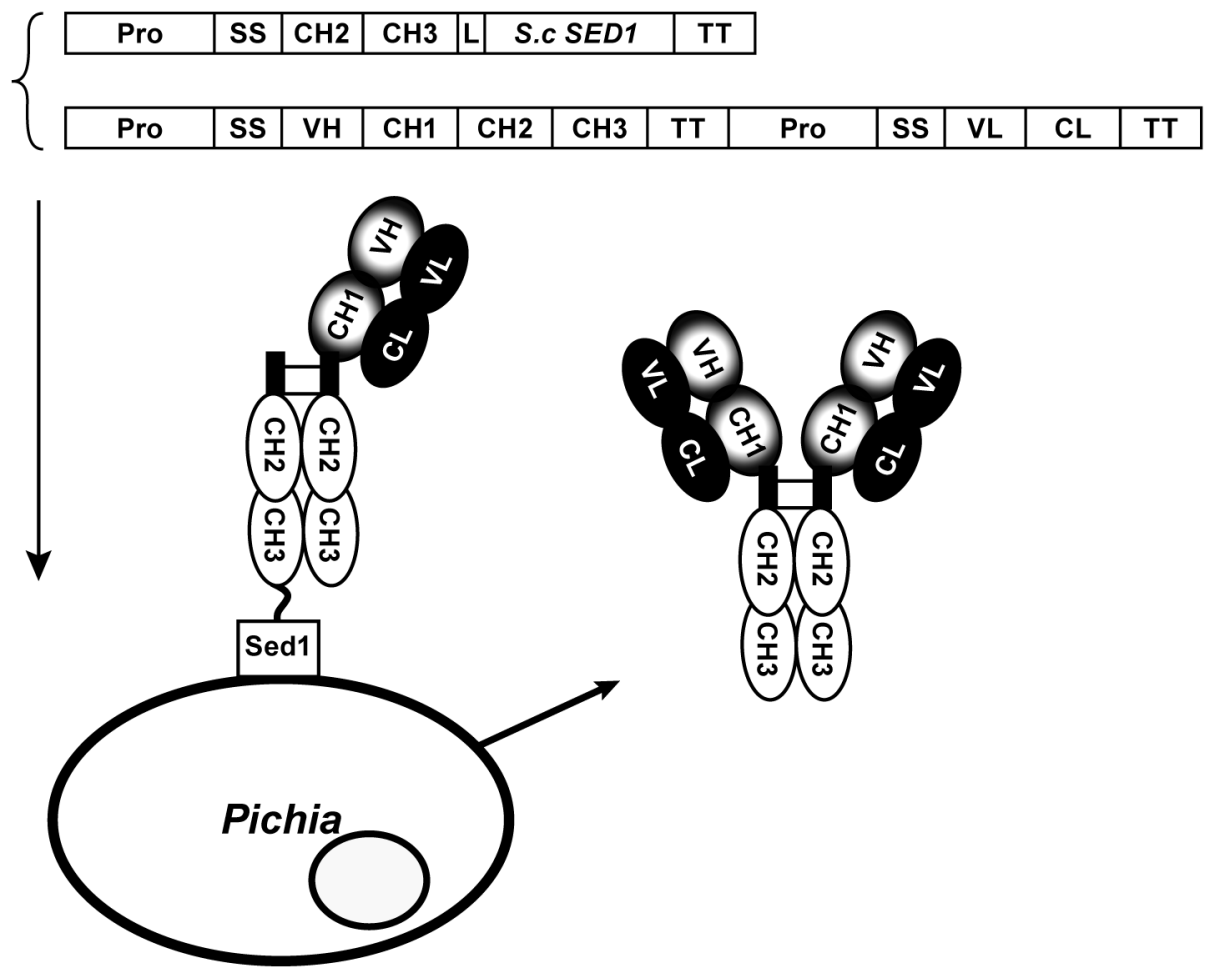
A schematic representation of the Fc-Sed1p antibody display system. The DNA sequence of the hinge region along with the CH2 and CH3 domains comprising the IgG Fc fragment are fused through a flexible linker to a cell wall anchoring partner, in this case the *S. cerevisiae* GPI anchor Sed1p is used. When co-expressed in the same host with a secretable full length IgG molecule, the Fc portion of the anchored fusion (the bait) heterodimerizes in the ER with the Fc region of the IgG molecule, forming two disulfide bridges. Since the CH1 domain of the heavy chain can still pair with the CL domain of the secreted light chain, this tripartite complex results in surface display of the monovalent (H+L) IgG molecule. Meanwhile the assembly of soluble full length IgG occurs with equal probability resulting in secretion of the bivalent (H2+L2) in the culture medium.

We designed a dual-mode antibody display system for presenting antibody molecules on the surface of *Pichia* *pastoris* . In this system, the hinge region along with CH2-CH3 domains comprised in the crystalizable fragment (Fc) of the IgG1 molecule was directly fused to the N-terminus of the previously described glycophosphoinositol (GPI) protein *Sc*Sed1p [3]. The introduction of this fusion in yeast results in the targeting of the Fc to the cell wall. As described in Figure 1, once this anchored fusion is co-expressed with a full-length IgG molecule, the Fc portion of the Fc-Sed1p fusion is expected to hetero-dimerize and form inter-disulfide linkages, through their respective hinge regions, in the endoplasmic reticulum (ER) with the Fc portion of the heavy chain (Hc) of the half IgG molecule. The complex interaction of Fc-*Sc*Sed1p-Fc(Hc+Lc) coupled to the association between the heavy chain and the light chain (Lc) within the antigen binding fragment (Fab) results in the display of the monovalent Hc+Lc moiety on the cell surface. The concomitant and independent association of the soluble heavy and light chain units of the full length IgG molecule would result in the secretion of fully assembled antibody molecules.

**Figure 2:**
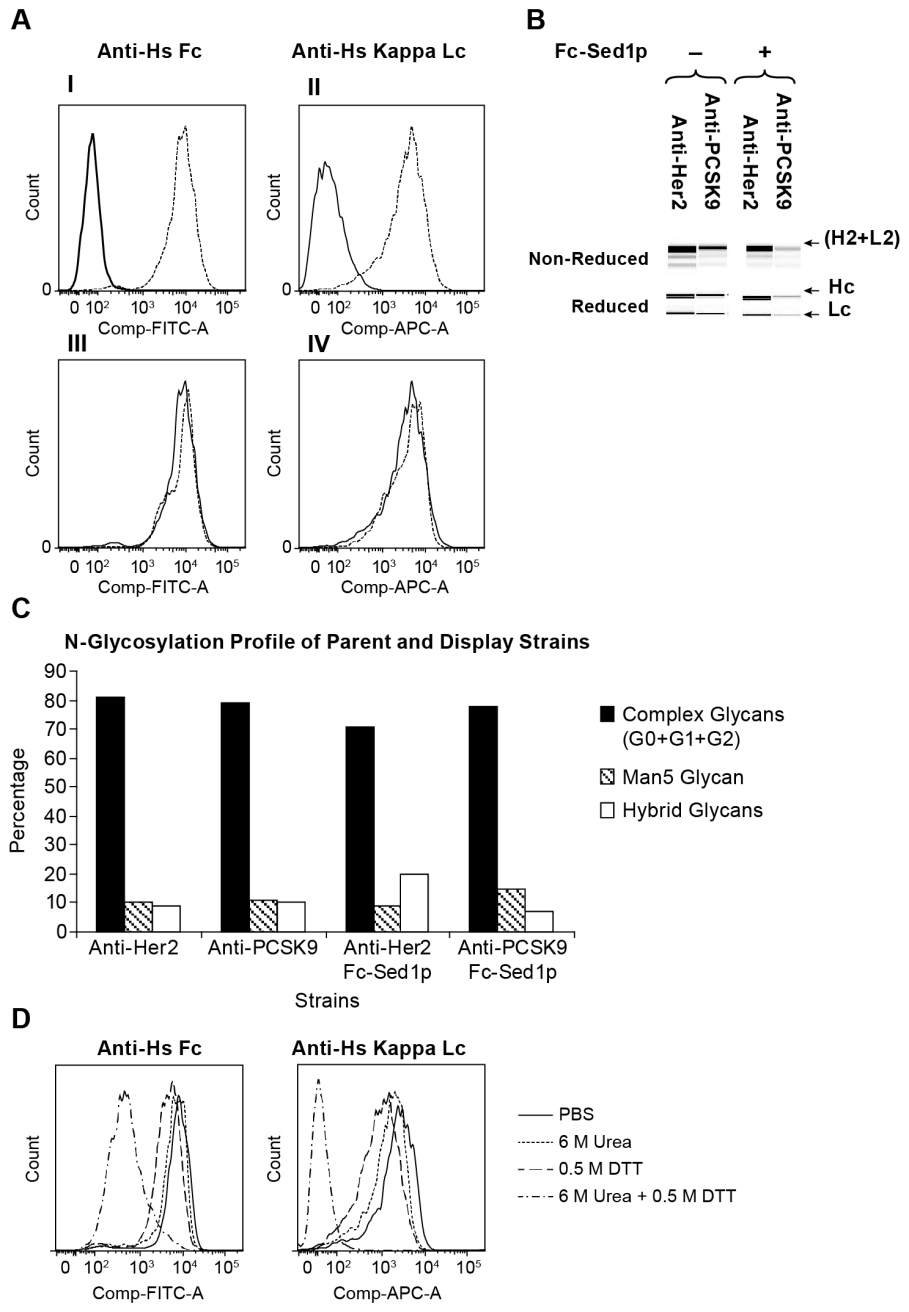
Efficiency of the surface anchored Fc bait (Fc-Sed1p) in capturing monovalent IgG molecules on the cell wall of Glyco-engineered *Pichia pastoris*. **A**) (I) Strain expressing soluble anti-PCSK9 antibody (solid histogram) and anti-PCSK9 strain co-expressing Fc-Sed1p fusion (dotted) were incubated with DyeLight 488 anti-Fc antibody and florescence intensities were assayed by flow cytometry (II). Same strains as in (I) were incubated with APC-conjugated anti-Kappa antibodies to detect light chain on cell surface (III). Flow cytometry was used to compare strain co-expressing anti-PCSK9 and Fc-Sed1p anchor (solid) against strain co-expressing anti-Her2 and Fc-Sed1p (dotted) for binding to DyeLight 488 anti-Fc or (IV) APC-anti Kappa. **B**) Detection of co-secreted full length IgG in antibody producing strains with and without Fc-Sed1p. Culture medium was passed through a protein A column, IgGs were eluted and protein was resolved in a native or reduced form through Protein Chip analysis **C**) Bar graph of percentage human N-glycosylation abundances of protein A purified mAbs isolated in **B. D)** Cells co-expressing anti-PCSK9 and Fc-Sed1p fusion were induced and incubated for 10 minutes at room temperature in 1XPBS buffer (solid); 1XPBS buffer containing 6 M Urea (dotted); 1XPBS buffer containing 0.5 M DTT (dashed); or 1XPBS containing 6 M Urea and 0.5 M DTT (complex). Cells were washed and labeled with anti-human Fc (I) (DyeLight 488) and anti-human Kappa Lc (II) (APC). Fluorescence intensities were analyzed by flow cytometry.

To reduce the above concept into practice, we introduced the Fc-*Sc*Sed1p expression cassette into three Glyco-engineered *Pichia* *pastoris* strains. As described in Table 1 these strains included yeast YGLY18483 which expresses full-length anti-human PCSK9, and YGLY13979 which expresses full-length anti-human Her2 IgGs. We assayed antibody display and expression in each strain following cultivation in 1L bioreactors by two independent methods. First, we examined the display of monovalent IgGs on the cell surface by monitoring the expression of the light chain on the yeast cell surface. Cells were labeled with anti-human IgG Kappa chain and their fluorescence intensity was analyzed using flow cytometry (Figure 2A). We reasoned that the presence of the light chain on the cell surface would be indicative of the successful hetero-dimerization and assembly of cell wall anchored Fc-*Sc*Sed1p and monovalent Hc+Lc IgG molecule. Second, the supernatants of cultures in (Figure 2A) were passed through a Protein A column to capture secreted full-length IgG. The eluents were subsequently resolved by electrophoresis to establish the presence of full-length fully assembled IgGs (Figure 2B). As demonstrated in Figure 2A (I; II), only strains containing both Fc-*Sc*Sed1p bait and full-length IgG expression cassettes reacted with anti-human Kappa light chain antibodies indicating the ability of the system to display the (Hc+Lc) half antibody molecules. Moreover, electrophoresis of Protein A-captured pools confirmed that the supernatants of the display strains contain levels of secreted full-length IgGs that are similar to those of the non-display parents without Fc-*Sc*Sed1p bait (Figure 2B). We also employed 2-aminobenzamide (2-AB) labeling and high performance liquid chromatography (HPLC) to analyze the glycan content on IgGs at Fc N297 site produced in strains with or without Fc-*Sc*Sed1p. We observed that the glycosylation signatures in the display-secretion strains (with "Fc bait") were similar to those of non-display parent strains without "Fc bait" (Figure 2C). Taken together these experiments prove that this system is capable of displaying monovalent antibodies while maintaining simultaneous secretion of soluble full length assembled (Hc2+Lc2) molecules. Furthermore, the secreted IgGs are modified with human-type N-glycans that are comparable to those that occur on molecules produced by the "non-display" strains.

To demonstrate that the robustness of dimerization between the "bait" Fc-*Sc*Sed1p and Fc region of IgG (Hc+Lc) occurs through hinge region disulfide linkages, we subjected cultures displaying anti-Human PCSK9 to various denaturation conditions and then probed the cell surface for the presence of IgGs. As described earlier, we labeled cells with fluorescently-conjugated anti-human Fc and anti-human IgG Kappa chain antibodies followed by flow cytometry. Only when cultures were treated with 0.5M DTT in the presence of 6M urea, did we observe a complete loss in the ability to detect the light chain on the surface of these cells (50% of Fc was still detectable due to the presence of cell wall-anchored Fc-Sed1p bait) (Figure 2D). We concluded that in this method, the capture of the half antibody (Hc+Lc) on the cell surface occurs via both hydrophobic and covalent interaction in a manner similar to the assembly of full length IgGs.

**Figure 3:**
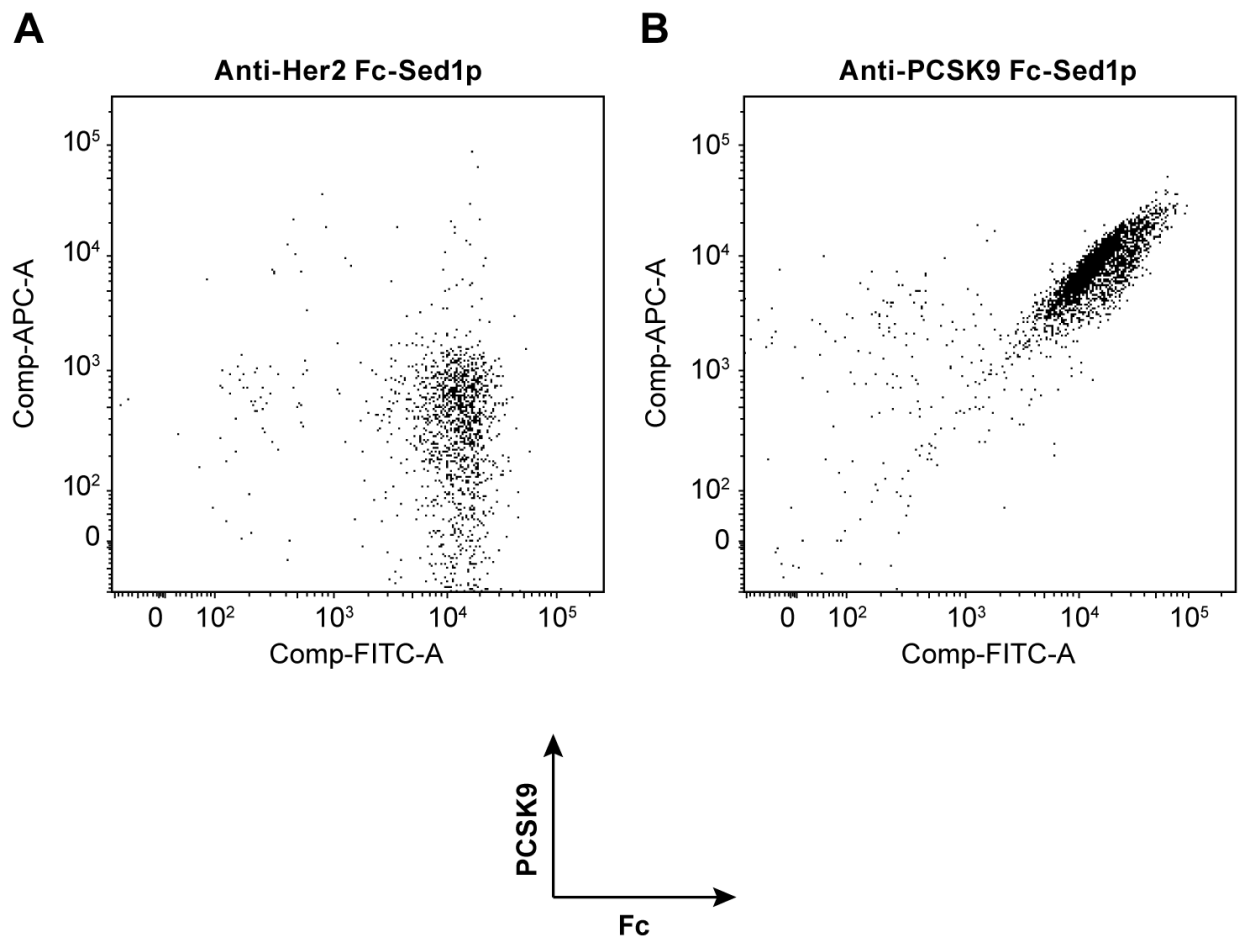
Antigen binding capacity of monovalent IgGs displayed by Fc-Sed1p measured by flow cytometry. The cells were dually labeled with goat anti-human Fc DyeLight 488, biotinylated PCSK9, and APC 635 labeled Streptavidin **A**) FACS analysis of labeled *Pichia pastoris* strains displaying Fc-Sed1p complexed with monovalent anti-Her2 antibody fragment (H+L) or **B**) an monovalent anti-PCSK9 (H+L) antibody fragment.

We next sought to establish whether the displayed monovalent antibodies are properly assembled and are able to bind to their cognate antigens. To this end, we probed the ability of displayed anti-PCSK9 (Hc+Lc) antibody to bind specifically to biotinylated human PCSK9. An anti-Her2 display strain was used as a negative control. We incubated both display strains with fluorescently labeled anti-human Fc and biotinylated human PCSK9. As shown in Figure 3A, yeasts displaying anti-Her2 on cell surface were able to bind to the anti-Fc reagent only, while cells displaying anti-PCSK9 reacted with both biotinylated PCSK9 antigen and anti-human Fc antibody (Figure 3B).

**Figure 4:**
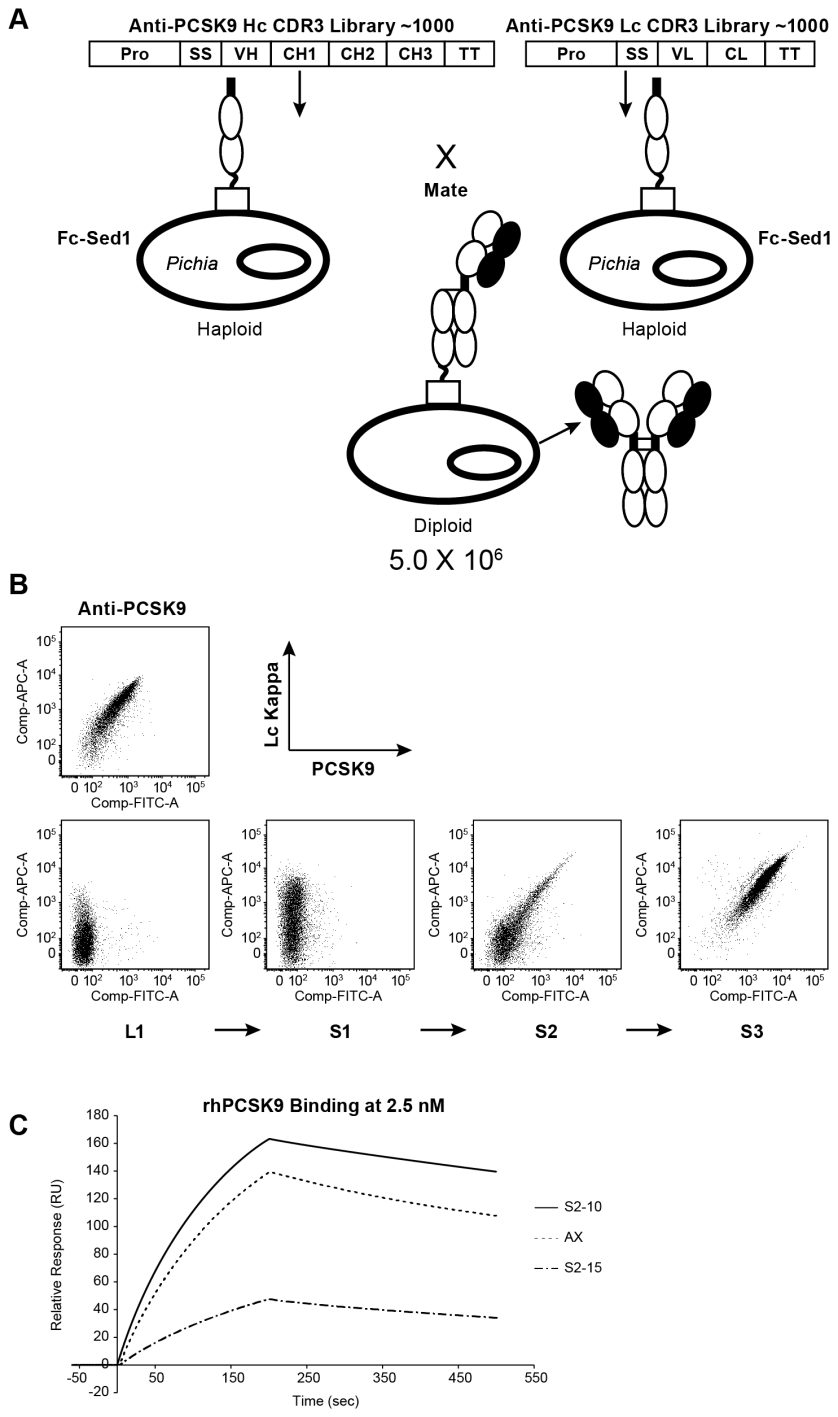
The construction of a proof of principle mating library and the isolation of high affinity, and high expression antigen binders using Fc-Sed1p. **A**) Haploid *Pichia pastoris* strains containing Fc-Sed1p expression cassette were transformed with a library of Anti-PCSK9 Hc or a library of Anti-PCSK9 Lc. Following mating and selection, diploids were cultured to express full length IgG library. Cultures were labeled using 20nM biotinylated human PCSK9 and APC 635 labeled antihuman Kappa. DyeLight 488 labeled Streptavidin was used to detect biotinylated PCSK9. **B**) Analysis and enrichment of high affinity anti-PCSK9 binders using three rounds of sequential sorting (S1 > S2 > S3). Known anti-PCSK9 strain co-expressing Fc-Sed1p was labeled as above as a control. **C**) Sensograms showing binding and dissociation kinetics of rhPCSK9 to immobilized anti-PCSK9 antibodies. Each anti-PCSK9 antibody lead was captured on the chip to ~ 500 RU followed by analyte injections of wild-type human PCSK9 at concentrations ranging from 0.156–2.5nM. Kinetics for the highest PCSK9 concentration (2.5 nM) is depicted for these antibodies.

The goal of developing this novel antibody surface display is its application in antibody discovery and maturation while preserving full-length IgG secretion. For that purpose, we designed a proof-of-concept study to demonstrate the utility of this display method in maturing the expressibility of an antibody lead in Glyco-engineered *Pichia* *pastoris* while maintaining the affinity to its known antigen. An anti-PCSK9 mAb (mAb1) was originally identified from a phage display library with low nano-molar affinity. In order to isolate novel anti-PCSK9 leads with optimal expression in Glyco-engineered *Pichia* *pastoris* , two synthetic DNA libraries covering heavy and light chain CDR3 regions were designed and synthesized by site directed mutagenesis. The final diversity of the heavy chain library was 4,320 while the final diversity of the light chain library was 1,296. Both heavy chain and light chain libraries were introduced separately into haploid *Pichia* *pastoris* strains containing the Fc-*Sc*Sed1p expression cassette. To create a full-length antibody library, haploid libraries of the heavy and light chains were combined by mating to generate a diploid full-length IgG library with a diversity of ~5 x 10^6^. We sequenced the heavy and the light chain genes from 40 diploid strains. This confirmed both the presence of heavy and light chains, and the amino acid sequence diversity in the library, as all of the clones tested contained unique antibody genes. We also analyzed affinities of secreted IgGs produced in these diploids by surface plasmon resonance (SPR). Although the majority of these diploid clones did not produce high levels of full-length antibodies as assessed by ELISA and SDS-PAGE (data not shown), no PCSK9 binders were observed in this randomly selected pool where full length IgG was produced, thus indicating majority of mutations that were introduced to this antibody resulted in loss of optimal expression and antigen binding (See Figure 4B, panel L1). We subsequently employed FACS to isolate highly-expressed mAbs that retained binding to PCSK9. To this end, three rounds of sorting were carried out. Twenty nM biotinylated PCSK9 antigen was used in each round, and the top 1% binders were isolated based on fluorescence intensity. After each round, ~1000 clones were plated on solid medium. Forty clones were selected, and their heavy chain and light chain genes were PCR amplified and sequenced to determine their amino acid sequence. Since this method enables simultaneous display and production of mAbs, leads selected by FACS were directly cultured in 96-well plates to produce full-length secreted IgG for *in vitro* PCSK9 binding studies.

As depicted in Figure 4B, when compared to the anti-PCSK9 positive control, we were able to enrich for a pool of positive PCSK9 binders following three rounds of FACS (Figure 4B: L1 > S1 > S2 > S3). Interestingly, the final round of sorting enriched a single mAb candidate (Figure 4B: S3), as suggested by gene sequencing data, this clone was highly represented after two sorting rounds which indicates the high efficiency of FACS enrichment (Table 2). Using single-point (2.5 nM rhPCSK9) SPR, the affinities of a select number of co-secreted IgGs from round two clones were determined. As listed in Table 2 we were able to isolate a novel mAb against PSCK9 with affinity similar to the original anti-PCSK9 lead. The anti-Kappa ELISA protein titers for the novel leads reached up to 30 µg/mL compared with 10 µg/mL for the mAb1 control.

**Table 2:** Analysis of anti-PCSK9 variants following 2^nd^ round of enrichment.

**Sequence ID**	**No of Clones of Identical Sequence**	**Average *KD* nM**	**ELISA Titer (µg/mL)**
S2-10	28	0.99±0.037	30.9±9.7
S2-15	11	60.75±15.46	26.2±9.3
S2-32	1	559.5	25.3
AX Parent	Control (n=8)	2.25	10.5±4.2

We sought to generate novel anti-PCSK9 binders with optimized expressibility in Glyco-engineered *Pichia*, while maintaining affinity of the original lead. To confirm this in a bioreactor setting, the selected clones: high affinity S2-10, and medium affinity S2-15 were cultivated and fermented in small-scale bioreactors to generate full-length mAbs. Originator Anti-PCSK9 (AX) expression strain was included as a control. Following fermentation, mAbs were purified using Protein A chromatography. The concentration of antibody was determined using Protein A HPLC analysis and affinities were measured by SPR by injecting rhPCSK9 at concentrations ranging from 0.156 to 2.5 nM. Figure 4C and Table 3 show that the ranking of affinities of these novel IgGs were in agreement with data obtained in 96-well experiment, however the affinity values for lead S2-15 appeared lower in the 96-well experiment (Table 2). This is primarily due to the nature of the biacore data that is obtained by assaying crude supernatants from 96-well plates using a single concentration of the antigen. For that reason this assay can only be used for highthroughput clone selection, and data from the purified samples (Table 3) are reported for the actual affinities.

**Table 3:** Binding Affinities and Titers of anti-PCSK9 variants.

**Sequence ID**	**No of Clones**	Average ka (1/M*s)	**Average kd (1/s)**	**Average KD (nM)**	**Highest Titer (mg/L)**
AX	2	2.04E+05	8.66E-04	4.32±09	100
S2-10	4	2.40E+05	5.10E-04	2.14±0.2	550
S2-15	4	1.65E+04	1.12E-03	7.17±2.1	480

The newly identified S2-10 exhibited a slightly slower “Off” rate but maintained almost identical “On” rate compared to the original molecule (AX), which might account for the difference in affinity (2.14 nM compared to 4.32 nM, respectively) (Table 3). Moreover, the expression levels of newly identified leads reached up to 0.5 g/L compared with ~100mg/L observed for the original anti-PCSK9 lead AX. This demonstrates that this display method is capable of discovering novel high affinity antibody leads with improved production titers.

We have presented the development of a novel dual-mode display and secretion technology in Glyco-engineered *Pichia* *pastoris* , and demonstrated the utility of this method in selecting mAb leads with variable affinities and high productivity. The combination of display and secretion in the same clone enables the continuity and fidelity of the antibody discovery process and could lead to shortened maturation cycle and desirable lead developability.

## Discussion

Here, we describe, a novel system for the display of monovalent functional antibodies on the surface of Glyco-engineered *Pichia* *pastoris* . This technology takes advantage of the antibody dimerization in the ER to achieve capture of the Fc region of a half mAb to an anchored Fc molecule through covalent disulfide bond formation in the hinge region, while preserving the assembly and secretion of free soluble full-length IgGs. Previous methods have utilized Fab anchoring through pairing of protein partners in the ER, where one of the monomers is anchored to a surface molecule. Examples of such display systems include the Aga1-Aga2 [27] and the GR1-GR2 [3] systems. The utilization of the native Fc region as an anchoring surrogate, nevertheless, simulates the process of antibody assembly, and thus eliminates expression or stability biases that could be introduced through ectopic anchoring sequences.

This surface display module in combination with FACS could be employed to screen large IgG libraries for high affinity and well-expressed leads that also possess desirable stability profilesYeast libraries could be subjected to various robustness tests to select clones that maintain antigen binding following denaturing conditions. N-glycans can impart desired biophysicochemical properties on proteins including solubility and formulability [28–31]. Antibody libraries in Glyco-engineered *Pichia* can be designed to include canonical glycosylation sites in the Fab region. FACS can be used to obtain sequences containing N-glycosylation that do not interfere with antigen binding affinity. In our studies, we used Glyco-engineered *Pichia* strains to establish proof of principle of the anchored Fc bait as a dual-mode antibody surface display and production platform. In a similar manner, it is conceivable that any eukaryotic antibody production cell line could be used to display monovalent (Hc+Lc) while simultaneously secreting full-length IgGs. Other membrane or cell wall proteins can be used to anchor Fc regions of any IgG subclass. However, Glyco-engineered *Pichia* offers advantages over other established antibody discovery platforms. It is able to generate IgG molecules with human glycoforms while maintaining the high transformation efficiency of yeast. Furthermore, the tractability of a fungal system enables co-evolving the cell line and molecule of interest in the same experiment to generate “genetically-customized” production hosts for each discovered lead. It is of importance to note that this system as well as other display formats may be biased towards selecting antibodies with lower affinity and higher expression over ones with higher affinity but lower expression. For that reason extra care should be employed in designing and performing FACS experiments to include clones with such criteria.

**Figure 5:**
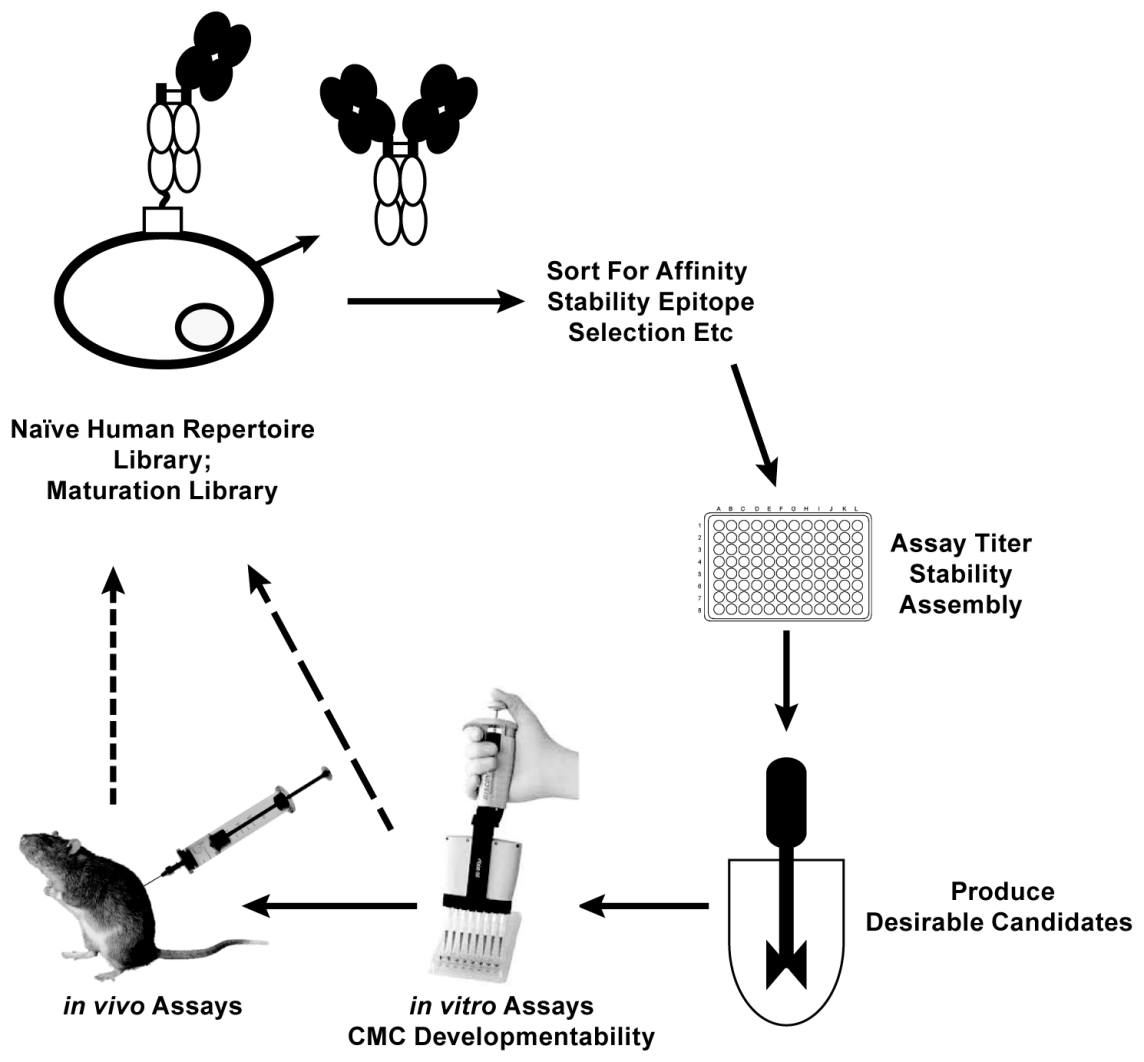
An antibody discovery and optimization scheme that utilizes Fc-Sed11p display-secretion technology in humanized *Pichia pastoris*. This paradigm enables progression from lead discovery to preclinical validation and development using a single host.

Surface display boasts the ability to link genotype to phenotype. In the classical antibody display paradigms, sorted clones are isolated and their genotypes are subcloned into new production hosts. This cloning step involves screening multiple clones to isolate ones with defined properties. The anchored Fc system side-steps the need for these additional steps by combining selection of antibody affinity and production host in a single experiment. Libraries can be generated and displayed in the cell line of choice, such as Glyco-engineered *Pichia* *pastoris* . Selected clones can be fermented directly in small or large scale vessels to generate material for *in vitro* assays such as affinity measurements, cell culture assays, or physicochemical properties. Successful candidates can be fed directly into *in vivo* preclinical models via simple scale-up production in stirred tank reactors (Figure 5). If desired, once a clone is selected, genetic approaches can be utilized to evict the anchored Fc cassette to generate an antibody production strain.

The development of a dual antibody surface display concept that preserves the ability to simultaneously secrete full-length IgG simplifies the process of therapeutic antibody discovery and development. It also has the potential to shorten development timelines and increase the probability of success. When combined with GlycoFi mAbs expression platform in Glyco-engineered *Pichia* *pastoris* , it provides a bona fide next-generation, end-to-end antibody discovery and production platform.
